# Clinical utility of evoked potentials for programming subthalamic deep brain stimulation in Parkinsons disease

**DOI:** 10.1038/s41531-026-01274-2

**Published:** 2026-01-23

**Authors:** Blake Hale, Anna Latorre, Lorenzo Rocchi, John Rothwell, Patricia Limousin

**Affiliations:** 1 Department of Clinical and Movement Neurosciences, UCL Queen Square Institute of Neurology, University College London, London, UK; 2 Department of Clinical Neurophysiology, National Hospital for Neurology and Neurosurgery, University College London Hospitals, Queen Square, London, UK; 3 Department of Medical Sciences and Public Health, University of Cagliari, Cagliari, Italy

**Keywords:** Diseases of the nervous system, Neurophysiology, Neurological disorders, Therapeutics

## Abstract

Optimal subthalamic nucleus deep-brain stimulation (STN-DBS) for Parkinson’s disease reduces motor symptoms without stimulating adjacent structures and causing side-effects. Fine-tuning STN-DBS using clinical evaluation is time-consuming and often requires multiple follow-ups. Electrophysiological recordings may enhance STN-DBS device programming for clinicians by providing objective evidence of neural pathway activation. This literature review critically evaluates evoked potentials as biomarkers of optimal STN-DBS and assesses potential integration into the device programming toolkit.

## Introduction

Over the past three decades, deep-brain stimulation (DBS) has made significant strides as a powerful treatment for refractory neurological and psychiatric disorders. DBS is a neurosurgical procedure that enables circuit-based neuromodulation by implanting electrodes in specific regions of the brain and delivering electrical pulses from an implanted battery source. Parkinson’s disease (PD) is one of the most common conditions treated with DBS. Although high-frequency subthalamic nucleus (STN)-DBS has been used for over 25 years to improve motor symptoms, the exact mechanism of its action remains poorly understood^1–3^. However, research suggests that STN-DBS effectively reduces excessive beta frequency oscillations in both the STN and sensorimotor cortex, and that this reduction correlates with improvements in PD motor symptoms, particularly bradykinesia^4–8^.

**Fig. 1:**
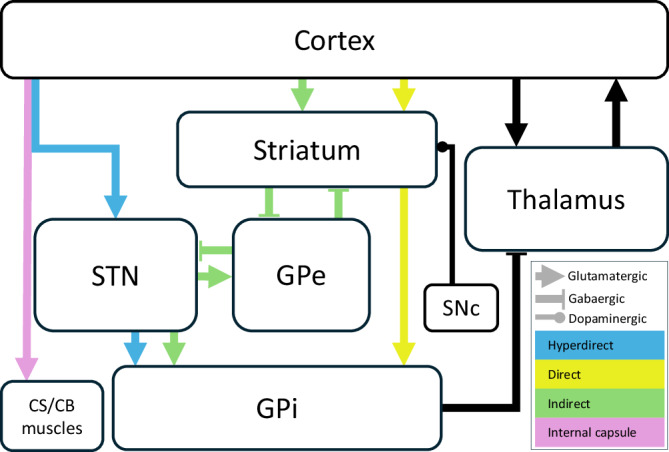
Basal ganglia and associated pathways. Schematic overview of basal ganglia circuitry and associated pathways, including the cortico–subthalamic (hyperdirect) pathway, relevant to mechanisms discussed in this review. *GPe* globus pallidus externus, *GPi* globus pallidus internus, *SNc* substantia nigra pars compacta, *CS/CB* corticospinal/corticobulbar (created based on descriptions and illustrations in Mtui and coworkers^12^).

One proposed mechanism underlying the effect of STN-DBS is the modulation of hyperdirect pathway (HDP) activity^9^. The HDP is composed of myelinated fibres that connect layer 5 cortical pyramidal cells and the STN (Fig. 1)^10–12^ and, in conjunction with the direct and indirect pathways, it shapes the dynamics of action selection and initiation. Unlike other basal ganglia pathways, the HDP is a monosynaptic connection from the frontal cortex to the STN, believed to provide rapid inhibition for action suppression^10,13,14^. Although the exact role of the HDP in PD is not fully elucidated, the relief of PD motor symptoms through STN-DBS has been associated with antidromic activation of the HDP and reduction in beta activity in both the cortex and the STN^5,7,9,15–24^.

The STN is a small, variably sized structure with an oblique orientation, surrounded by a complex network of pathways and nuclei, necessitating accurate targeting during DBS procedures^25,26^. Following surgical implantation, DBS programming in the clinical setting aims to identify parameters that optimally stimulate the STN while avoiding unintended interactions with neighboring structures^27–29^. Currently, the standard approach for selecting the optimal contact, stimulus amplitude, frequency, and pulse duration involves post-surgical imaging and trial-and-error clinical assessments^28,30,31^. The traditional DBS electrode array of four evenly spaced, cylindrical, omnidirectional ring contacts has been predominantly supplanted by arrays with tripartite segmented contacts that allow for current steering and directional refinement^28,32^. As DBS systems become increasingly sophisticated, tailoring programming sessions to maximize therapeutic benefit while minimizing side effects is becoming increasingly complex. Additionally, the variability in patient symptoms—particularly in those taking PD medication—and the potential delay in both beneficial and adverse effects further complicate the programming process. Together, these factors can make identifying the optimal settings a time-consuming process, often requiring multiple clinic visits^33–35^.

**Fig. 2:**
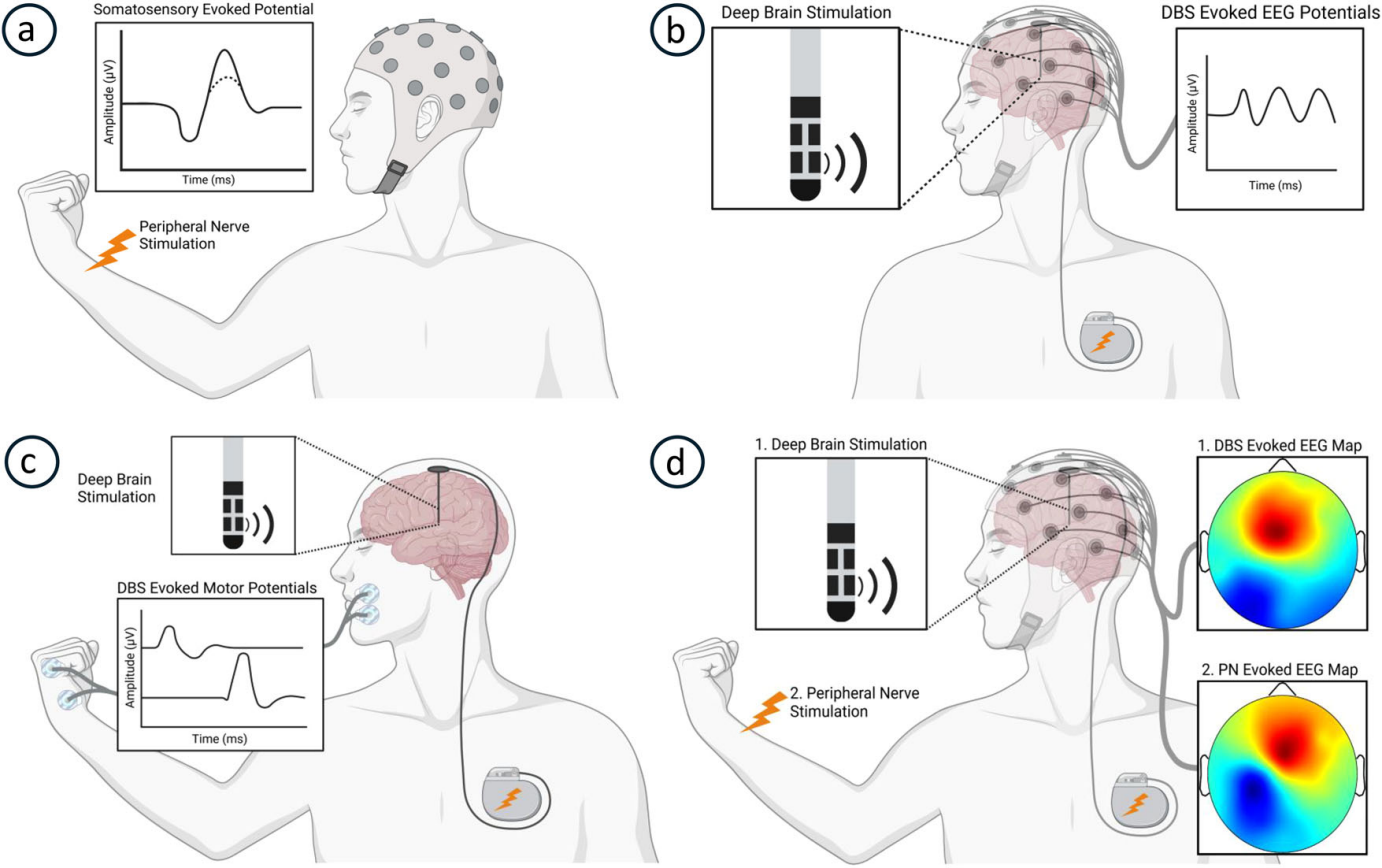
STN-DBS evoked potential recordings. Diagrams of electrophysiological methods to evidence accurate STN-DBS for Parkinson’s disease therapy. **a** The negative potential 30 ms after peripheral nerve stimulation may be attenuated in Parkinson’s disease (dotted line) and restored with accurate STN-DBS. **b** Cortical STN-DBS evoked potentials recorded with scalp electrodes can correlate with clinical efficacy. **c** STN-DBS evoked muscle activity recorded with skin surface electrodes can demonstrate unwanted internal capsule stimulation to predict motor side effects. **d** Scalp voltage maps evoked by STN-DBS similar to a somatosensory evoked potential topography produced by contralateral peripheral nerve stimulation could demonstrate unwanted ipsilateral medial lemniscus stimulation and predict somatosensory side-effects. *PN* peripheral nerve. *Created in BioRender. Hale, B. (2025)* https://BioRender.com/5wdnmoz.

An emerging approach to DBS leverages the recordable potentials elicited in the STN and surrounding structures to enhance reliability and efficiency of device programming. STN-DBS evoked potentials provide an objective means of correlating stimulus parameters with activation of neural pathways in the outpatient setting (Fig. 2). Additionally, these evoked potentials can help physiologically localize STN-DBS electrode contacts and their corresponding stimulus fields. This review aims to evaluate the role of evoked potentials as a non-invasive, post-implantation method for defining the therapeutic window and optimizing STN-DBS parameter selection. In the first section, we examine the literature on sensorimotor evoked potentials and STN-DBS cortical evoked potentials (cEPs) as biomarkers of STN-DBS efficacy. The second section explores how motor and somatosensory evoked potentials (MEPs and SEPs, respectively) may serve as biomarkers for the cardinal side-effects of STN-DBS. We then discuss practical considerations before proposing future directions toward meaningful implementation of these electrophysiology investigations as programming tools. Throughout this review, we assess the utility of evoked potentials in localizing DBS contacts within the basal ganglia and refining the programming of STN-DBS for PD therapy.

## STN-DBS Efficacy Biomarkers

### Median nerve N30 evoked potential

Somatosensory deficits in PD include impairments in proprioception^36,37^, tactile perception^38–40^, sensory attenuation^41–43^, and temporal^44–46^ and spatial discrimination^40^. Such deficits can disrupt sensorimotor integration and contribute to PD symptoms^41,42,47,48^. Scalp electrode recordings of cortical potentials evoked by electrical stimulation of peripheral nerve afferents have been used to investigate somatosensory dysfunction in PD. At rest, the primary somatosensory cortex response recorded over the centroparietal scalp 20 ms (N20) after median nerve stimulation is typically unaffected in PD^49–51^. In contrast, the frontal component observed 30 ms after stimulation (N30) is often reduced or absent^50–55^ (Fig. 2a). This abnormality may reflect deficient dopaminergic tone and impaired output from the basal ganglia to the supplementary motor areas and primary motor cortex associated with PD^56–58^. Notably, administration of dopamine agonists^54,59,60^, pallidotomy^61^, and both globus pallidus interna (GPi) and STN-DBS can restore the N30 potential in PD patients^52,62^. Furthermore, N30 amplitude has been negatively correlated with rigidity and the Unified Parkinson’s Disease Rating Scale motor scores^51,59^, with therapy-induced amplitude increases proportional to clinical improvements^59^. This suggests that restored N30 amplitude could serve as a biomarker of STN-DBS efficacy.

However, the N30 is not a universally accepted biomarker of sensorimotor dysfunction. Some studies report no selective N30 amplitude reduction in PD^49,63^, while others have found that motor improvements following dopaminergic therapy do not always correlate with N30 increases^64,65^. Additionally, changes in N30 amplitude linked to therapy occur gradually: Pierantozzi and colleagues^52,62^ allowed 4 hours to achieve effective STN-DBS stimulation, and N30 amplitude decayed over 60 min after its withdrawal. These time-dependent changes present methodological challenges for future studies and limit the practical advantages of using N30 amplitude as a clinical biomarker for STN-DBS efficacy.

### STN-DBS cortical evoked potentials

While DBS for PD targets specific anatomical subregions within the basal ganglia, its clinical effects result from broader alterations in brain network dynamics. Studies delivering transcranial magnetic^66^ and direct electrical stimulation^67,68^ of the motor cortex intraoperatively elicit recordable STN potentials, which may guide accurate lead placement in the motor subregion. However, this technique is not viable in the current clinical programming context because once DBS hardware is internalized, freely recording from STN electrodes is not possible due to vendor software constraints^67,68^. On the other hand, cortical activity elicited by STN-DBS can be studied using electrocorticography (ECoG), EEG (Fig. 2b), and magnetoencephalography (MEG). Though studies are technically challenging (as discussed in section 4.0), several reports using sub-therapeutic stimulus rates have revealed antidromic and orthodromic cortical activities time-locked to STN-DBS, some of which have been mapped to specific neural pathways^17,20,69,70^. While researchers have categorized these responses based on latency ranges, inconsistencies persist — some studies report onset latencies, while others focus on peak latencies, which may be negative, positive, or unspecified due to electrode referencing differences. To align with the majority of the literature and facilitate comparison, we categorized cEPs into early (<2 ms), short (2–15 ms), and long-latency (>15) responses. This categorization also seeks to account for methodological variability across studies, as presented in Table 1 and discussed below.

**Table 1:** Publications until August 2024 recording human evoked cortical potentials in the context of STN-DBS for PD

Publication	Stimulation Paradigms	Cortical Recording (sampling frequency; bandpass)	Early peaks (<2 ms)	Short-latency peaks (2–15 ms)	Long-latency peaks (>15 ms)
Limousin et al.^130^	STN-DBS: single	EEG (not stated): frontal, central	Not investigated	Not investigated	N18-20
Postoperative, awake^	Polarity not stated	Ref: recorded bipolar, not stated how analysed but bipolar inferred			
Ashby et al.^16^	STN-DBS: single, paired, trains	EEG (not stated; 2-1 kHz): frontopolar, frontal, central, parietal	Not investigated	N3, N5, N8, P10, N12	Not investigated
Postoperative, awake^	Bipolar	Ref: recorded unipolar (ipsilateral ear), analysed unipolar (ipsilateral ear) and bipolar			
Baker et al.^15^	STN-DBS: single, paired, burst	EEG (600 Hz, 4 kHz; 0.05-250 Hz): frontal, central, parietal	P1-2	N8, P14	Single, paired stimulation: N29, P70, N160
Postoperative, awake or asleep*	Bipolar	Ref: recorded unipolar (linked mastoid reference) and bipolar, not stated how analysed but unipolar and bipolar inferred			Burst stimulation: P149, N230, P320
MacKinnon^78^	STN-DBS: single	EEG (2 kHz; 30-1 kHz): frontal, central, parietal	Not investigated	Ventral contact stim: 12.5 ± 2.2 frontal (P) parietal (N)	Ventral contact stim: 16.8 frontoparietal phase reversal
Postoperative, awake*	Bipolar (monopolar abandoned due to artefact)	Ref: recorded unipolar (contralateral mastoid), not stated how analysed but unipolar inferred			Dorsal contact stim: 23.2 ± 3.7 frontal (P) parietal (N)
	Median nerve (for scalp topography)				
Eusebio et al.^8^	STN-DBS: trains	EEG (1.5 kHz; 0.25-300 Hz): 10:20 array (London), sensorimotor areas (Marseille)	Not investigated	3-8 (inconsistent and disregarded due to stimulus artefact)	21.2 ± 1.3 (London) 21.6 ± 0.6 (Marseille)
Postoperative, awake^#^	Bipolar	Ref: recorded unipolar (linked ears), not stated how analysed but unipolar inferred			
Kuriakose et al.^133^	STN-DBS: single	EEG (5 kHz; 5-500 Hz): frontal, central	Not investigated	~N3, ~N7	P22.7 ± 1.7
Postoperative, awake*	Bipolar	Ref: recorded unipolar (linked ears), not stated how analysed but unipolar inferred			
Walker et al.^17^	STN-DBS: single	EEG (not stated): frontal, central	P1.0 ± 0.4	P5.7 ± 1.1	P22.2 ± 1.8
Postoperative, awake	Bipolar	Ref: not stated but unipolar inferred as contralateral mastoid mentioned for analysis			
Miocinovic et al.^20^	STN-DBS: single	ECoG (22 kHz; 1-10 kHz): premotor, primary motor, primary sensory, superior parietal lobule	P1.5 ± 0.1	P2.8 ± 0.3, P5.8 ± 1.0 (at M1), P7.7 ± 1.8 (all areas)	N20 ± 8, P38 ± 11, N56 ± 15, P71 ± 16
Intraoperative, awake	Bipolar and monopolar	Ref: recorded unipolar (ipsilateral scalp or ear), analysed bipolar			(P and N termed ‘peaks’ and ‘troughs’)
	Median nerve (to identify M1)				
Hartmann et al.^110^	STN-DBS: single	MEG (5 kHz; 10-250 Hz): whole-head	~P1	P4 ± 0, P11 ± 1	N27 ± 6
Postoperative, awake	Bipolar				
Romeo et al.^71^	STN-DBS: single	EEG (25 kHz, 20 kHz; 1–10 kHz): 64-electrodes	0.86 ± 0.09	High frequency activity onset: 9.0 ± 0.3	High frequency activity onset: 9.0 ± 0.3, lasting for ~25 ms
Intraoperative and postoperative, awake	Bipolar	Ref: recording not stated but unipolar inferred as “re-referenced” to bipolar for analysis			
Irwin et al.^82^	STN-DBS: single	EEG (50 kHz; 1–10 kHz): 12 or 64-channel	~N0.5	R2-R3 (N and P peaks 6-30), suppressed with anaesthesia	R2-R3 (N and P peaks 6-30), suppressed with anaesthesia
Intraoperative, awake and anesthetised^	Bipolar	Ref: recording not stated but unipolar inferred as “re-referenced” to bipolar for analysis			
Chen et al.^13^	STN-DBS: single	ECoG (22 kHz; 0.075–3.5 kHz): rostral middle frontal, inferior frontal gyrus, superior temporal	Not investigated	N2.2 ± 0.2, P6.0 ± 0.35	Not investigated
Intraoperative, awake	Bipolar	Ref: recorded unipolar (ipsilateral scalp vertex or linked mastoid), not stated how analysed but unipolar inferred			
Levinson et al.^81^	STN-DBS: trains 180 and 185 Hz	ECoG ( ~ 6 kHz; low-pass 200 Hz): primary motor cortex	Not investigated	Not investigated	P61 ms after stimulus train start
Intraoperative, anesthetised*	Bipolar	Ref: recorded and analysed unipolar using unspecified scalp electrode			P45 ms after stimulus train stop
Howell et al.^109^	STN-DBS: single	ECoG (22 kHz; 0.075-3.5 kHz): primary motor cortex	~P1.5	P2-4	Not investigated
Intraoperative, awake	Bipolar and monopolar	Ref: recorded unipolar (ipsilateral unspecified scalp or ear), analysed bipolar			
Sand et al.^173^	STN-DBS: single	EEG (1 kHz; 0.5–40 Hz): 64-channel	Not investigated	Not investigated	P62 with dorsolateral STN stim.
Postoperative, awake	Monopolar	Ref: recorded unipolar (Cz), analysed average reference			
Bahners et al.^108^	STN-DBS: single	MEG (not stated): motor cortex, SMA, IFG, MFG	Not investigated	P5.6, P11.8	P21.8
Postoperative, awake*	Monopolar				
Campbell et al.^80^	STN-DBS: symmetric biphasic, conditioning pulse	EEG ( ~ 24 kHz; 8-100 Hz): extended 10-20	Not investigated	~P2, P and N 10-150	P and N 10-150
Postoperative, awake*	Bipolar and monopolar	Ref: recording and analysis not stated			
Spooner at al.^137^	STN-DBS: single	MEG (5 kHz; 0.1–1660 Hz): whole-head	Not investigated	3–10, expressed as < 10	16-26, expressed as ~20
Postoperative, awake	Monopolar				
Peeters et al.^76^	Directional STN-DBS: single	EEG (16,382 Hz; low-pass 3.2 kHz): 64-channel	Not investigated	P3, P10	Not investigated
Postoperative, awake	Multiple independent controlled current STN-DBS: single	Ref: recorded and analysed unipolar (Cz)			
	Monopolar				
Peeters et al.^75^	Directional STN-DBS: single	EEG (16,382 Hz; low-pass 3.2 kHz): 64-channel	Not investigated	P3, P10	Not investigated
Postoperative, awake	Monopolar	Ref: recorded and analysed unipolar (Cz)			
Peeters et al.^34^	Directional STN-DBS: single	EEG (16,382 Hz; low-pass 3.2 kHz): 64-channel	Not investigated	P3, P10	Not investigated
Postoperative, awake	Monopolar	Ref: recorded and analysed unipolar (Cz)			
Peeters et al.^77^	Directional STN-DBS: single	EEG (16,382 Hz; low-pass 3.2 kHz): 64-channel	Not investigated	P3, P10	Not investigated
Postoperative, awake	Monopolar	Ref: recorded and analysed unipolar (Cz)			
Peeters et al.^74^	Directional STN-DBS: single	EEG (16,382 Hz; low-pass 3.2 kHz): 64-channel	Not investigated	P3, P10	Not investigated
Postoperative, awake	Monopolar	Ref: recorded and analysed unipolar (Cz)			
Borgheai et al.^101^	STN-DBS: single	ECoG (22 kHz; 0.075–3.5 kHz): primary motor cortex (*note: STN-DBS local evoked potential also recorded*)	cathodic: 1.5 ± 0.2	cathodic: 2.7 ± 0.4, 4.8 ± 0.5, 6.5 ± 0.8	Not investigated
Intraoperative, awake	Monopolar: cathodal and anodal with ipsilateral shoulder return	Ref: recorded unipolar (linked earlobes), analysed bipolar	anodic 1.7 ± 0.3	anodic: 2.8 ± 0.4, 5.0 0.5, 7.0 0.9	
Spooner et al.^24^	STN-DBS: single	MEG (5 kHz; 0.1-1660 Hz): whole head	Not investigated	3–7	Not investigated
Postoperative, awake*	Monopolar				

*P* positive polarity, *N* negative polarity (polarity undefined if not indicated), *M1* primary motor area, *SMA* supplementary motor area, *IFC* inferior frontal cortex, *MFG* middle frontal gyrus, *stim* stimulation, *Ref* reference montage(s) used for recording and analysis. Subjects were OFF Parkinson’s disease medication except when ON*, ON and OFF^#^, or not stated^.

#### Methodologies

Addressing the methodological heterogeneity of the studies in Table 1 is essential for assessing clinical translation. Stimulation modalities were monopolar, bipolar, both, or unspecified. Monopolar stimulation, the most common clinical DBS mode, is often the default because it simplifies programming^71–73^, making it preferable for translational research^71^. However, in the 25 reviewed cEP studies, bipolar stimulation was slightly more frequent (44%) than monopolar (40%), with five monopolar contributions from Peeters and colleagues^34,74–77^). Bipolar stimulation is often chosen to reduce artifact^20,71,78,79^; for example, Mackinnon and colleagues^78^ used it because monopolar stimulation contaminated cEPs in the first 30 ms. Yet both modes can produce artifacts^7,24^ and appropriate techniques can mitigate these issues (see Section 4.0). Miocinovic and coworkers^20^ found that both modalities evoke comparable cEPs on ECoG, and monopolar stimulation may even yield more robust short-latency cEPs at lower intensities^80^.

Although six of the 25 studies were intraoperative, 88% of all recordings were performed with subjects awake. Under general anesthesia, Levinson and colleagues^81^ reported long-latency cEPs, whereas Irwin and coworkers^82^ found 6–10 ms peaks present in wakefulness were abolished. This underscores the need to control for anesthesia. In the report by Baker and colleagues^15^, subjects “typically slept” during recording; since STN activity^83,84^ and associated PD biomarkers fluctuate during sleep^84^, this may affect the reliability of their cEPs.

Recording techniques also varied (Table 1), and no study directly compared cEPs across modalities. EEG was used in 64% of studies, likely due to its low cost, non-invasiveness, and broad availability^85,86^. MEG, increasingly used in STN-DBS cEP research, offers millimeter spatial resolution^87–89^ versus the centimeter range of EEG^90,91^, while both provide millisecond temporal resolution^86,91^. However, replication of cEP findings between EEG and MEG is lacking. Comparisons between EEG and the 20% of intraoperative ECoG studies should also be cautious: while both measure neuronal electrical activity, ECoG strips are closer to the cortex, offering millimeter resolution and higher signal-to-noise ratios^92–95^. Burnos and colleagues^96^ studying median nerve stimulation, found EEG potentials were delayed by ~0.3 ms compared to ECoG, but no direct comparisons exist for STN-DBS stimulation.

Differences in recording and analysis configurations across ECoG, EEG, and MEG may further limit generalizability. Unlike MEG, which measures absolute magnetic fields^97^, EEG and ECoG record voltage differences between active and reference electrodes. Montages are typically unipolar, with references positioned far from the brain region of interest^98^ to boost signal amplitude^98–100^, but Table 1 shows wide variability (e.g., linked mastoids, ipsilateral ear, Cz) and unclear locations in several studies^13,17,71,80,82^. Different reference positions can significantly alter recorded signals^98,99^. Offline referencing also varied: ECoG studies used unipolar and bipolar equally; EEG most often used unipolar (9/16 studies), with Cz the most common site (6/9), likely for its distance from motor areas yet relative artifact resistance. While amplitudes vary considerably^15,75,81^, latencies are generally used for assigning biophysical processes and for cross-subject generalization. How non-standardized referencing impacts latency comparisons remains unclear, but—along with the other methodological variations—should be considered when classifying cEP latencies

#### Early cEPs

Studies recording cortical activity, with adequately high sampling rates and effective stimulus artefact reduction, have reported that the earliest cEPs evoked by STN-DBS peak at latencies of less than 2 ms. This early cEP is likely due to antidromic activation of the internal capsule (IC) corticospinal/bulbar tract fibers, rather than HDP activation (Fig. 1)^20,82,101^. This hypothesis is supported by several lines of evidence. The IC is composed of large, myelinated fibers that typically run parallel to the stimulating electrode and are often located some distance from it. Because these corticospinal/corticobulbar tract axons pass by the electrode, their activation is governed by the second spatial derivative of the extracellular potential^101,102^. The very short-latency EP is consistent with this mechanism and aligns with the known corticospinal conduction velocity of ~40 m/s and the estimated 6 cm distance from the primary motor cortex (M1) to the IC^20,103^. This relationship is further supported by evidence from non-human primates, where antidromic pyramidal tract conduction velocities of at least 46 m/s have been reported^104^. Over distances of 6.0–7.5 cm, these velocities yield response latencies in the range of 1–2 ms, reinforcing the plausibility of the observed human cEP timing^20,105,106^. Similarly, according to computational models, the conduction velocity required to generate such short-latency responses would exceed that of HDP fibers activated near the STN^107^. In addition, early cEPs are localized over M1, whereas short-latency cEPs (2 – 15 ms), which are more clearly linked to activation of the HDP, exhibit a broader cortical distribution^16,20,82,108^. Further supporting this, electromyography (EMG) recordings from muscles receiving projections from the corticobulbar and corticospinal tracts have revealed EMG bursts time-locked to the early cEP^16,20,82,101^, presumably due to activation of fibers in the IC^82^. Accordingly, Howell and coworkers^109^ attributed cEPs peaking around 1.5 ms to antidromic activation of the corticospinal/corticobulbar tracts, even without recording concurrent EMG. Furthermore, cEPs can also be evoked by GPi-DBS, which lacks a monosynaptic cortical connection but is adjacent to the IC^20,70^. Taken together, these findings strongly indicate that the early cEP arises from IC rather than HDP activation^20,70,107,110^.

In suitable DBS systems or before leads are internalized, it is possible to record local field potentials from DBS contacts adjacent to those delivering STN-DBS. Here, unlike the low frequency (1-20 Hz) stimulation used in cEP studies, high-frequency (70-180 Hz) pulse bursts are employed, producing and modulating evoked resonant neural activity (ERNA) in the STN^111,112^. Some ERNA features have been correlated with clinical efficacy^111–116^. Given that STN-DBS evokes measurable potentials from the STN itself, some researchers propose that the early cEP is actually a local STN potential volume-conducted to the scalp^117^. However, there is scant evidence supporting this theory. Stimulus artifact contamination is prominent in ERNA under 1 ms^113,118,119^, and most studies focus on post-stimulus oscillations occurring 4–20 ms after stimulation^120,121^. Although a few studies with adequate signal processing have reported ~0.3 ms STN potentials, they did not concurrently record EEG, leaving the relationship between STN potentials and the early cEP unclear^115,122^. Furthermore, ERNA is highly sensitive to stimulus parameters^111,122^ and the low-frequency stimulation used in cEP studies may not produce the same ERNA observed with high-frequency burst stimulation. Future research incorporating simultaneous STN recordings, EEG, and improved stimulus artifact reduction in the sub-1 ms period will be critical in clarifying the origins of the early cEP and its potential role in optimizing STN-DBS therapy.

#### Short-latency cEPs

Activation of the ipsilateral HDP and disruption of beta synchronization between the STN and cortex are considered key mechanisms underlying the therapeutic effects of STN-DBS^9,13,17,20,71,123,124^. Although computational models^125^ and primate HDP studies offer valuable insights^126^, detailed anatomical and biophysical data on the human HDP are still lacking^127^. Based on animal studies, the HDP is described as primarily composed of thin collaterals branching from corticofugal axons that traverse the internal capsule and continue towards the brainstem — although some axons may form direct projections^126,128,129^. A recent study demonstrated that HDP activation with STN-DBS involves axonal elements distinct from the large, myelinated fibers that pass distantly from the electrode. Instead, activation likely occurs through stimulation of passing axons at varying distances, as well as terminal axons synapsing within the STN^101^. Despite these anatomical uncertainties, imaging and electrophysiological evidence strongly support the presence of EEG potentials within the 2–15 ms latency as marker of HDP activation^13,20,34,70,74,75^. Notably, the HDP connects the cortex to the STN but not to the GPi (Fig. 1); accordingly, short-latency cEPs are present with STN-DBS but absent with GPi-DBS^20,70,130,131^. These findings suggest that short-latency cEPs—particularly the ~3 ms peak (P3)—may serve as reliable physiological markers of HDP activation^20,101^, and potentially of STN-DBS efficacy.

Ashby and coworkers^16^ first identified a frontal cortical potential peaking at ~3 ms (P3) that was maintained at 100 Hz stimulation without evidence of blocking. Given these biophysical properties, they hypothesized that the P3 arose from a synapse-free circuit and involved the activation of fast-conducting, myelinated axons connecting the STN and cortex^16^. Although the same authors initially observed P3 following stimulation of more ventral contacts^16^, the underlying structure was later identified as the HDP^10,132^. Subsequent studies using EEG^15,17,34,74,75,78,133^, ECoG^20,69,70,109^ and MEG^110^ have further implicated the dorsolateral located HDP in the generation of these short-latency responses. In parallel, MRI tractography studies have also provided strong evidence of widespread HDP connections between the frontal cortex and the ipsilateral STN^9,14,134,135^, with HDP tract activation correlating with reductions in PD symptoms, thereby identifying so-called STN-DBS “sweet-spots”^9^.

Other studies lend further support to the P3 as biomarker of HDP activation and also of therapeutic STN-DBS efficacy. First, STN-DBS contacts closer to the dorsolateral STN and HDP produce the highest P3 amplitudes^75^, and the same wave was found to be larger over the ipsilateral primary motor area^20^. Ashby and coworkers^16^ reported contacts evoking short-latency cEPs were later used clinically, but provided few details. Subsequent studies have demonstrated that P3 amplitude strongly correlates with contacts delivering PD motor symptom therapy^20^, as well as higher side-effect thresholds and wider STN-DBS therapeutic windows^34,74–77^. Miocinovic and colleagues^20^ demonstrated monopolar stimulation of longer pulse width produced larger short-latency cEPs with unchanged latencies, however they did not investigate clinical correlates. These findings support the hypothesis that the short-latency P3 component represents antidromic HDP activation from accurate STN subregion stimulation, positioning it as a promising biomarker for STN-DBS efficacy^34,75,76^.

Previous STN-DBS studies have also reported cEP peaks at 8–10 ms when stimulating with contacts located lateral^71^ or ventral^16,78,133^ to the optimal STN target. Romeo and coworkers^71^ identified an ~8 ms peak correlated with clinical motor side effects, though the underlying mechanism was not explored. However, it seems unlikely to reflect corticospinal tract activation because the latency would be 1-2 ms, given the biophysical properties described for early cEPs. Peeters and colleagues found that increasingly ventral stimulation produced larger amplitude peaks at ~10 ms^74–76^, which inversely correlated with side-effect thresholds^34^, suggesting activation of the substantia nigra pars reticulata (SNr) and SNr-premotor tracts^74^. Here, unlike in the study by Romeo and colleagues^71^, side-effects were not subdivided into motor or non-motor^34^. Future investigations into the neurophysiology underlying 8–10 ms latency peaks and their association with side effects may further establish the utility of these early cEPs as biomarkers for optimizing therapeutic outcomes in STN-DBS.

#### Long-latency cEPs

Given cortex-STN latencies in primates are 5-6 ms^136^, potentials with prolonged onsets (>15 ms) are generally considered inconsistent with monosynaptic, antidromic HDP conduction^8,78^. Instead, they are more likely to result from orthodromic stimulation of the polysynaptic basal ganglia-thalamocortical circuit^15,17,20,70,78,108,130,137^. Fifteen of the 25 studies in Table 1 report these potentials, having been recorded on ECoG, EEG, and MEG. Mackinnon and colleagues^78^ found no strong correlation between the optimal clinical stimulation contact and the largest amplitude long-latency cEP over the sensorimotor area (23 ms peak latency). In contrast, Spooner and coworkers^137^ with MEG and Kuriakose and colleagues^133^ with EEG reported contacts producing ~20 ms and 22 ms peaks correlated with clinically beneficial contacts, respectively. The exact neural substrates generating these long-latency cEPs remains speculative. However, some researchers propose that this activity—especially that up to 400 msec reported by Baker and colleagues^15^—represents polysynaptic cortico-basal ganglia-thalamo-cortical reverberant loops, possibly sustained by the pathological PD neural substrate^15,69^. Further research elucidating the neurophysiological correlates and establishing clinical utility of long-latency cEPs is warranted.

### STN-DBS side-effect biomarkers

While STN-DBS is an effective treatment for fluctuating PD motor symptoms and levodopa-induced dyskinesia^138,139^, overstimulation can itself induce dyskinesia^140^. This side-effect is generally managed by reducing PD medication or reprogramming the STN-DBS device^141^. Notably, stimulation contacts that initially induce dyskinesia during programming may actually help identify the optimal therapeutic contact^142,143^. The most common and troublesome side-effects arise from unintended stimulation of structures adjacent to the STN subregion target, such as the pyramidal tract and ascending somatosensory pathways, and include muscle contractions and paresthesias^26,28,144,145^. Avoiding these tracts presents a common challenge in optimizing STN-DBS for PD motor symptom relief^146^. While some degree of side effects may be unavoidable, advancements such as current steering and segmented directional contacts have expanded the therapeutic window of STN-DBS. However, these refinements also add complexity to the selection of optimal stimulation parameters^25,33,147^. Recording MEPs and SEPs may not only help maximize clinical benefits but also aid in detecting subclinical thresholds for side effects. Table 2 provides an overview of research utilizing MEPs and SEPs in the context of STN-DBS.

**Table 2:** Publications until August 2024 measuring the effects on motor and sensory pathways in humans of STN-DBS for PD

Publication	Stimulation Paradigms	Brain Recording (sampling rate; bandpass)	Muscular Investigation (sampling rate; bandpass)	Somatosensory Investigation
Ashby et al.^150^	STN-DBS: single, paired, burst	Not investigated	MEP (2 kHz; 100–1 kHz): facilitated and present ( < 27 ms) and inhibition ( ~ 50 ms) – FDI, averaged	Not investigated
Postoperative, awake	Bipolar, monopolar (cathodal)			
Ashby et al.^16^				
Postoperative, awake^	STN-DBS: single	EEG (not stated; 2-1 kHz): frontopolar, frontal, central, parietal	MEP (not stated; 2–1 kHz): facilitated ( < 25 ms) and inhibition – FDI, averaged	Not investigated
	Bipolar, monopolar (cathodal)	Ref: recorded unipolar (ipsilateral ear), analyzed unipolar (ipsilateral ear) and bipolar	EEG: early cEP not detected (stimulus artefact noted), short-latency cEPs attributed to hyperdirect pathway	
Klosterman et al.^163^	Median nerve at wrist	STN electrode (10 kHz; 5–428 Hz)	Not investigated	No recordable potential at STN, far-field thalamic potentials only
Intraoperative, anesthetised^		Ref: recorded and analysed bipolar		
Hanajima et al.^164^	Median nerve at wrist	STN electrodes (12–15 kHz; 500–2.5 kHz)	Not investigated	Thalamic activity volume conducted to STN recording, possibly medial lemniscus activity
Post-operative, awake^		Ref: recorded unipolar (unspecified non-cephalic) and bipolar, analysed bipolar		
Hanajima et al.^162^	Median nerve at wrist	STN electrodes (2–2.5 kHz)	Not investigated	No recordable potential at STN, volume conduction from thalamus
Intraoperative and postoperative^, awake^		Ref: recorded unipolar (unspecified non-cephalic) and bipolar, analysed bipolar		
MacKinnon^78^	STN-DBS: single	EEG (2 kHz; 30–1 kHz): frontal, central, parietal	MEP (2 kHz; 30–1 kHz): at rest - Biceps, FCR, TA, averaged (absent).	Scalp: 14 ± 3 ms and N23 ± 4 ms
Postoperative, awake*	Bipolar, monopolar (cathodal)	Ref: recorded unipolar (contralateral mastoid), not stated how analysed but unipolar inferred	EEG: early cEP not detected (stimulus artefact noted)	
	Median nerve at wrist (for SEP scalp topography)			
Eusebio et al.^8^	STN-DBS: single	EEG (1.5 kHz; 0.25–300 Hz): frontopolar, frontal, central, parietal	Clinical assessment (n/a)	Not investigated
Postoperative, awake^#^	Bipolar	Ref: recorded unipolar (linked ears), not stated how analysed but unipolar inferred		
Kuriakose et al.^133^	STN-DBS: single Paired transcranial magnetic (TMS; 2–5 ms post-DBS)	EEG (5 kHz; 5–500 Hz): frontal, central	MEP (5 kHz; 20–2.5 kHz): facilitation with TMS - FDI (19.7 ± 0.99 ms latency), averaged	Not investigated
Postoperative, awake*	Monopolar (cathodal)	Ref: recorded unipolar (linked ears), not stated how analysed but unipolar inferred	EEG: early cEP not detected (stimulus artefact noted)	
Mahlknecht et al.^35^	STN-DBS: single	Not investigated	MEP (10 kHz; 20–1 kHz): at rest and facilitated - orbicularis oris, FDI, averaged	Not investigated
Postoperative, awake*	Monopolar (polarity not stated), variable pulse-width in subset of subjects			
Miocinovic et al.^20^	STN-DBS: single	ECoG (22 kHz; 1–10 kHz): premotor, primary motor, primary sensory, superior parietal lobule	MEP (22 kHz, 1019 kHz): at rest - nasalis, genioglossus trapezius, bicep, FCR, FCR, FDI, TA, (locations varied between subjects), unaveraged sweeps	Scalp median SEP for subdural electrode localisation
Intraoperative, awake	Monopolar, bipolar	Ref: recorded unipolar (ipsilateral scalp or ear), not stated how analysed but unipolar inferred	EEG: early cEP ( ~ 1.5 ms) associated with MEP	
	Median nerve (to identify M1)			
Shils et al.^148^	Directional STN-DBS: train of five	Not investigated	MEPs (not stated): at rest (anesthetized) - orbicularis oris, orbicularis occuli, Deltoid, Tricep, Biceps, ECU, FCU, FDI, APB, single sweep following train of stimuli	Not investigated
Intraoperative, anaesthetised^	Monopolar (cathodal)			
Nikolov et al.^149^	Directional STN-DBS: train Microelectrode: train of five	Not investigated	MEP (not stated; 0.5–2 kHz): At rest - orbicularis oris, FDI, averaged	Not investigated
Intraoperative, anaesthetised (1/15 was awake)	Bipolar (DBS cathode, ipsilateral temporal anode)			
Howell et al.^109^	STN-DBS: single	ECoG (22 kHz; 0.075–3.5 kHz): primary motor cortex	EEG: early cEP recorded ( ~ 1.5 ms) from contacts in posterolateral STN, proximal to internal capsule	Not investigated
	Bipolar, monopolar	Ref: recorded unipolar (ipsilateral unspecified scalp or ear), analyzed bipolar		
Intraoperative, awake				
Campbell et al.^80^	STN-DBS: symmetric biphasic, conditioning pulse	EEG ( ~ 24 kHz; 8–100 Hz): 10–20, 10-10	MEP (24 kHz; not stated): at rest - deltoid, bicep, tricep, FCR, EDC, averaged	Not investigated
	Monopolar (cathodal, anodal), bipolar	Ref: recording or analysis not stated	EEG: early cEP not detected (stimulus artefact noted), short-latency cEPs ( ~ 2 ms) attributed to hyperdirect pathway	
Postoperative, awake*				
Borgheai et al.^101^	STN-DBS: single	ECoG (22 kHz; 0.075-3.5 kHz): primary motor cortex	MEP (22 kHz; not stated): at rest - nasalis, orbicularis oris, biceps, FCR, ECR, FDI (cathodic: 11.7 ± 3.0, anodic: 12.0 ± 4.7), averaged	Not investigated
Intraoperative, awake	Monopolar (cathodal and anodal with ipsilateral shoulder)	Ref: recorded unipolar (linked earlobes), analyzed bipolar	EEG: early cEP recorded at 1.5 ms (cathodal) and 1.7 ms (anodal)	

*APB* abductor pollicis brevis, *ECU* extensor carpi ulnaris, *ECR* extensor carpi radialis, *EDC* extensor digitorum communis, *FDI* first dorsal interosseous, *FCR* flexor carpi radialis, *TA* tibialis anterior. Subjects were OFF Parkinson’s disease medication except when ON*, ON and OFF^#^, or not stated^.

### Methodologies

Heterogeneity in stimulation and recording techniques for side-effect assessment is also evident in Table 2. For IC activation, stimulation has been monopolar, bipolar, or both; polarity was reported in all studies except Mahlknecht and colleagues^35^. Campbell and coworkers^80^ and Borgheai and colleagues^101^ alternated polarities, highlighting the influence of pulse characteristics on IC activation. Most studies tested awake subjects, except the work by Shils and coworkers^148^ and Nikolov and colleagues^149^ who, unlike other studies using single or paired pulses, applied stimulus trains, a protocol uncommon in clinical practice. Recordings have not always included both EEG and MEPs; for example, Eusebio and coworkers^8^ used EEG with clinical assessment only. When measured, early cEPs were not consistently present alongside MEPs^16,78,80,133^, possibly due to artifact contamination and limited sampling rates (1.5 kHz^8^, 2 kHz^78^, 5 kHz^133^ or not stated^16^).

Muscle recordings uniformly used a belly–tendon montage, ensuring basic comparability, though muscle selection varied. All studies except Mackinnon and colleagues^78^ and Campbell and coworkers^80^ recorded from the first dorsal interosseous; these two, along with the reports by Ashby and colleagues^16,150^, did not assess corticobulbar-innervated muscles. Most studies averaged MEPs, while ~30% relied on reproducible single trials to confirm IC activation. MEPs in awake participants were recorded at rest or during facilitation via transcranial magnetic stimulation of M1 or voluntary contraction. The utility of using the active motor threshold was demonstrated by Mahlknecht and colleagues^35^. Overall, variations in stimulation—some not clinically relevant—and in recording approaches limit data generalization, though individual studies offer valuable insights for future work.

Investigations of unintended medial lemniscus activation have followed two approaches. The first, using peripheral nerve stimulation with intracranial recording, is methodologically flawed (see below). The second, adapting the method by Mackinnon and coworkers^78^, presents a novel opportunity to evaluate non-target activation; thus, no direct methodological comparison is possible.

### Motor evoked potentials

Motor side-effects of STN-DBS are among the most common^137^ and often the first to emerge^151^, and include oculomotor abnormalities, facial and limb muscle contractions which may cause dysarthria and impairment in motor function and gait disturbances^28,139,145,151–153^. The dorsomedial aspect of the IC converges on the rostro-ventral portion of the STN^154^, and its myelinated fibers are highly susceptible to unintended stimulation from STN-DBS current spread. The diffusion of STN-DBS current to the IC can lead to orthodromic pyramidal tract activation, resulting in motor side effects^25,35,133,152^.

Evoked potential recordings during device programming offer two key methods for detecting unwanted IC activation. As discussed in Section 1, the early cEP likely represents antidromic IC activation and could be utilized during DBS device programming^16,20,82,101,109^. For instance, Howell and coworkers^109^ found contacts located at the posterolateral STN elicited the largest amplitude early cEP at ~1.5 ms. Additionally, some studies have recorded EMG activity evoked by low frequency STN-DBS in cranial and limb muscles as markers of pyramidal tract engagement by STN-DBS, to refine STN targeting in chronically implanted patients^80,150,155^ (Fig. 2c). Cylindrical contacts in traditional STN-DBS arrays are located at varying distances from the IC, such that different monopolar or bipolar stimulation configurations may elicit MEPs of different amplitudes^20,35^. This enables functional localization of STN-DBS contacts, allowing clinicians to avoid those with a higher propensity for IC activation. Newer, non-cylindric directional contacts deliver more accurate stimulus fields, thereby reducing the risk of unintended IC fiber activation^148,151^. Additionally, other parameters such as stimulus polarity^101,156^, pulse geometry^80^, and current steering^25^ can significantly impact the ability to prevent pyramidal tract activation and associated side-effects.

Muscle contractions during device programming unequivocally identify unsuitable STN-DBS contacts. While clinical assessment of muscle twitching during device programming is useful for detecting IC activation^157^, even careful visual inspection may fail to identify subtle stimulation^35^. This highlights the need for objective electrophysiologic measurements to minimize motor side effects. In this context, the most useful clinical application of MEPs obtained with STN-DBS is their ability to predict motor side effects that may emerge after the programming session. EMG recordings can in fact reveal subtle muscle activation at rest during STN-DBS, which might otherwise go unnoticed^35,150,155^. Furthermore, voluntary muscle activation reduces the STN-DBS threshold required to elicit muscle responses^35^. This suggests that motor side effects, exacerbated by increased spinal motor neuron excitability, may not be apparent when a patient is relaxed during programming but could manifest later during daily activities. Incorporating STN-DBS MEP recordings into programming sessions would provide valuable insight into subclinical IC activation, enabling clinicians to optimize electrode selection and minimize unintended motor side effects.

### Somatosensory evoked potentials

A common side-effect of STN-DBS is paresthesia^28,158–160^. As the STN has no direct input to the somatosensory cortex, paresthesia likely arises from unintended stimulation of the medial lemniscus (ML), which lies ventroposteriorly to the STN^28,139,155^. In mild cases, paresthesias may be tolerable or habituate over minutes, days, or longer^139,161^. However, the persistence or emergence of troublesome paresthesia may necessitate usage of more dorsal DBS contacts^28^. While subjective patient reports are often sufficient for identifying problematic stimulation, it can take hours or even days for patients to recognize or report paresthesia that warrants device reprogramming. Despite the clinical relevance, few studies have explored the use of SEPs to detect STN-DBS current diffusion to the ML and predict the onset of paresthesia.

One approach attempted in prior research involves recording from STN-DBS electrodes during electrical stimulation of the contralateral median nerve during the implantation phase. Theoretically, the presence and amplitude of a signal detected at a given STN-DBS contact could serve as markers of proximity to the ML. However, action potentials within the ML are extremely small, and recordings primarily capture volume-conducted post-synaptic thalamic potentials rather than direct ML activation^162–164^. Additionally, implementing this method would require substantial modifications to DBS devices and software, making it impractical for postoperative programming.

A more feasible alternative is scalp EEG. Mackinnon and coworkers^78^ investigated somatosensory tract stimulation by STN-DBS using scalp EEG topography evoked by median nerve stimulation as a spatial reference for activation of the primary somatosensory area. They proposed that STN-DBS contacts producing EEG topographic maps similar to those evoked by median nerve stimulation indicate sensorimotor cortex activation, consistent with pallido-thalamic tract stimulation. This technique could also be applied to assess latent paresthesia during post-surgical programming. In this application, ML activation could be inferred if short-latency EEG topographies (~ 6 ms) evoked by STN-DBS closely resemble median SEP topographic maps at the typical 20 ms latency (Fig. 2d). STN-DBS contacts eliciting similar signals would then be identified and avoided in clinical usage. This approach offers a promising avenue for improving STN-DBS programming and minimizing sensory side effects, particularly for patients who experience delayed recognition of stimulation-induced paresthesia.

### Practical considerations

The preliminary studies reviewed above show that evoked responses hold some promise for improving DBS programming. However, practical clinical implementation will require standardization of both data collection and analysis methods. Of the techniques studied (EEG, ECoG, MEG), EEG is the most well-established in STN-DBS device programming studies (Table 1). ECoG is less susceptible to artifacts, has superior signal-to-noise ratio and excellent spatial and temporal resolution, but the invasiveness and localized recording only directly under the implanted ECoG strip limits usage^20,137,165^. In contrast, MEG offers whole-head, non-invasive, high-quality recordings but availability is limited by infrastructure demands, cost, and specialist training^97,137,166^. The emergence of the optically pumped magnetometers may alleviate some of these constraints^137,167^. From a technical standpoint, cEPs using EEG is currently the most appropriate technique for post-surgical STN-DBS device programming and we focus on EEG for the remainder of this section.

### Electrophysiological recordings

Despite the fact that the scalp N30 evoked by median nerve stimulation is easy to record^168–170^ and can be implemented in most clinical neurophysiology departments using existing equipment and personnel, there is no consensus on its utility as a biomarker of DBS efficacy. Indeed, the prolonged STN-DBS washout time limits clinical implementation.

More promising are the potentials evoked by stimulation through DBS electrodes themselves. There are a number of technical constraints which could limit application, but these can probably be overcome in most clinical neurophysiology departments. For example, to prevent temporal overlap between evoked cortical potentials, the stimulation rate has to be reduced below the usual ~130 Hz used clinically^15,16,78^. Fortunately, most current DBS devices support such low stimulation frequencies (5–20 Hz). Capturing early and short latency cEPs evoked by STN-DBS also requires recording equipment with high-sampling rates (typically >16 kHz in the literature), although these are quickly becoming standard in electrophysiology.

In addition to activity recorded from the scalp, it is important also to record EMG activity from facial and limb muscles in order to detect inadvertent activation of corticofugal motor fibers in the internal capsule. Although evoked potentials are conventionally recorded at rest, a separate session during voluntary contraction of target muscle is recommended. This is because STN-DBS MEP thresholds may be 30-35% lower during voluntary activation than at rest^35^, so that the chances of detecting activation are much higher. Motor side-effects are also more likely to manifest during activities of daily of life.

To assess unintended ML activation, a separate recording of the median nerve SEP template should be performed. Crucially, the stimulation frequency must match that used in the STN-DBS evoked potential phase to allow direct comparison of the evoked scalp voltage map.

In summary, most standard EEG and EP systems fulfil the specifications described above and make clinical DBS programming a feasible and promising step toward improving therapeutic outcomes. Signal analysis is currently performed offline; however, implementing real-time or near-real-time analysis pipelines would significantly enhance clinical utility by enabling immediate feedback during DBS programming sessions.

### Signal processing

One major challenge is discerning biological signal from the prominent STN-DBS stimulus artifact. While short pulse-widths and bipolar stimulation can help reduce artifact amplitude^20,71,78^, these parameters often do not reflect typical clinical stimulation settings^71^. One simple method is to utilize the common mode rejection of the differential amplifier and re-reference the recording to bipolar for analysis^71,82^. Anode-cathode reversal during recordings also reduces stimulus artefact. This method alternates monopolar stimulation using an STN-DBS contact as cathode half of the time and anode for the remainder, causing the stimulus artifact to invert and thus average out over successive stimuli while phase-locked neural signals are preserved^15,17^. However, this technique is flawed because different neural elements can be activated by different polarities of stimulation^20,80^.

To reduce contamination of biological signals by stimulus artefact, Peeters and coworkers^34^ applied an artifact template subtraction technique^171^. This takes an average of the stimulus artefact from electrodes unlikely to detect time-locked biological signals (e.g., linked mastoids) and subtracts that template from recordings over the motor cortex containing the putative biomarker. A limitation is that the distant electrodes record a slightly different artefact than the target electrode^172^, which contaminates the biological signals of interest. This could be addressed like Levinson and colleagues^81^ for ECoG who employed unsupervised dictionary-based artifact rejection that comprises automatically detected artifact templates from different electrodes^172^.

Stimulus artefact reduction using linear interpolation over a pre and post-stimulus period is another typical pre-processing method employed^34,74–77,81,173^. Unlike the subtraction method, this does not attempt to remove any artefact that overlaps the biological signal; it simply tidies up the visual display by removing a large spike in the record. Epochs containing excessive non-stimulus artefacts (e.g., eye-movements, mains interference) may be removed visually, by automatic detection, or both.

Post-acquisition signal processing is an evolving landscape of techniques and there is no consensus on the methodology for STN-DBS EPs. Analyses require specialist practitioners and computer programs which are widely available. Many papers utilize toolboxes within the MATLAB environment, such as the opensource EEGLAB^82,173–176^. One popular algorithm used to process signal is the ICA algorithm which disentangles overlapping cEPs from the multi-channel brain recording and separates them from each other and artefacts. A similar technique, principal component analysis (PCA), again decomposes the varied cEPs contained across all electrodes but assembles them into a few main patterns that explain most of the signal’s variance^177,178^. Reducing a complex cEP into its main patterns with PCA first may allow subsequent ICA to quickly and more accurately isolate neural sources^178^.

### Patient experience

Discomfort experienced due to withholding PD medication is equivalent to standard monopolar review but EP-based testing offers improved time-efficiency and less demand on patients. Electrodes applied with conductive paste or gel present only minimal cosmetic inconvenience whilst electrical stimulation of the median nerve for the SEP template rarely produces discomfort.

### Future directions

Establishing cEPs, MEPs, and SEPs as objective physiological biomarkers of STN-DBS efficacy is a valuable goal but is contingent on validation through real-world outcomes. Given the association of P3 with HDP activation and PD motor symptom reduction, short-latency cEPs represent the most promising candidate biomarkers of accurate and therapeutic STN-DBS. Though Peeters and coworkers^74^ encourage cautious interpretation of their findings regarding P3 they propose their P3-hotspot mapping technique could be integrated onto patient specific imaging for clinical programming. Future advances could produce an abridged stimulation protocol informed by clinical assessment, imaging, or LFP data. Though focused on the P3, other short-latency and long-latency cEPs could be included in the same analysis in order to maximize the available information content of the signal.

Given its high signal-to-noise ratio, ECoG data provides the most detailed information on the time course and location of cortically evoked activity (if sufficient spatial coverage is available). However, practical considerations mean that this has to be substituted by scalp EEG data in a clinical setting. Future work should therefore clarify the correspondence between intracranial ECoG signals and scalp EEG. Simultaneous recording of ECoG and EEG in a small number of cases—as in related studies^93,96^—could clarify this and enable direct comparison of cEP latencies and distributions between modalities. Similarly, recording MEG with simultaneous EEG is important to compare latencies between these modalities. This will become particularly important as MEG becomes a more accessible way to study STN-DBS neuromodulation.

Although this paper focuses on the application of evoked response methodologies, future clinical implementation is likely to be multimodal and incorporate other leading techniques such as image-guided programming and local field potentials (LFPs). For example, using image-guided programming, CT and MRI data of electrode position is combined with electric-field-based volumes of tissue activated models to predict the lowest-threshold sites of activation^179–181^. This can be used to identify the best electrodes to activate HDP, which could then be verified with P3 recordings.

Selection of the most effective contact for STN-DBS using LFPs can streamline initiation of therapy^182–185^ and closed-loop adaptive technology offers optimized chronic therapy^186–189^. Beta band metrics are the predominant LFP biomarkers, however beta frequencies are symptom, state, and task-specific^190,191^ with beta oscillations accounting for only a modest proportion of motor symptoms^192,193^. In addition, LFPs identify contacts for motor symptom benefit but lack information on side effect inducing off-target current spread^183^. Furthermore, LFP-guided therapy is constrained by the limited availability of sensing enabled hardware and specialist patient-specific expertise to interpret signals^188,194–196^. In contrast, evoked potentials leverage existing EEG systems and expertise, with cEPs detecting on-target stimulation and MEPs and SEPs directly evidencing unintended capsular and lemniscal activation. Evoked potentials therefore provide complimentary and additional information to LFPs and offer potential to enhance STN-DBS programming.

It is envisioned that EPs could be integrated with other programming optimization approaches during the typical week-long inpatient phase following STN-DBS implantation. Localizing STN-DBS contacts using these objective physiological markers at this earlier time may more accurately determine the optimal stimulus parameters, prevent trial-and-error follow-up appointments and enhance clinical outcomes.

## Conclusion

Advancing DBS technology increasingly enables clinicians to stimulate the STN with greater accuracy. However, the growing complexity of device programming, fluctuating symptomatology, and delayed clinical effects of STN-DBS pose significant challenges. Evoked potentials recorded non-invasively offer a promising avenue to guide electrode choice in order to optimize therapy, and reduce side effects during clinical programming sessions. Data collection requires minimal modification of standard clinical neurophysiology techniques and should be readily available in most hospital settings.

Among short-latency cEPs, the P3 component, which is indicative of HDP activation, has emerged as the most promising biomarker for STN-DBS efficacy whereas the primary side effects of STN-DBS—muscle contractions and paresthesia—can be minimized by monitoring MEPs and SEPs, respectively. Further work may clarify the potential roles of other short latency and long-latency cEPs to guide clinical programming.

## Data Availability

No datasets were generated or analysed during the current study.
